# The JcWRKY tobacco transgenics showed improved photosynthetic efficiency and wax accumulation during salinity

**DOI:** 10.1038/s41598-019-56087-6

**Published:** 2019-12-23

**Authors:** Prashant More, Parinita Agarwal, Priyanka S. Joshi, Pradeep K. Agarwal

**Affiliations:** 1 Plant Omics Division, CSIR-Central Salt and Marine Chemicals Research Institute (CSIR-CSMCRI), Council of Scientific & Industrial Research (CSIR), Gijubhai Badheka Marg, Bhavnagar, 364 002 Gujarat India; 2 Academy of Scientific and Innovative Research (AcSIR), Ghaziabad, 201002 India

**Keywords:** Metabolomics, Photosynthesis, Abiotic

## Abstract

Salinity is one of the major factors negatively affecting crop productivity. WRKY transcription factors (TFs) are involved in salicylic acid (SA) mediated cellular reactive oxygen species homeostasis in response to different stresses, including salinity. Therefore, the effect of NaCl, NaCl + SA and SA treatments on different photosynthesis-related parameters and wax metabolites were studied in the *Jatropha curcas* WRKY (JcWRKY) overexpressing tobacco lines. JcWRKY transgenics showed improved photosynthesis rate, stomatal conductance, intercellular CO_2_ concentration/ambient CO_2_ concentration ratio (Ci/Ca ratio), electron transport rate (ETR), photosynthesis efficiency (Fv/Fm), photochemical quenching (qP), non-photochemical quenching (NPQ) and quantum yield of PSII electron transport (ΦPSII) in response to salinity stress, while exogenous SA application had subtle effect on these parameters. Alkane, the major constituent of wax showed maximum accumulation in transgenics exposed to NaCl. Other wax components like fatty alcohol, carboxylic acid and fatty acid were also higher in transgenics with NaCl + SA and SA treatments. Interestingly, the transgenics showed a higher number of open stomata in treated plants as compared to wild type (WT), indicating less perception of stress by the transgenics. Improved salinity tolerance in JcWRKY overexpressing tobacco transgenics is associated with photosynthetic efficiency and wax accumulation, mediated by efficient SA signalling. The transgenics showed differential regulation of genes related to photosynthesis (*NtCab40*, *NtLhcb5* and *NtRca1*), wax accumulation (*NtWIN1*) and stomatal regulation (*NtMUTE*, *NtMYB*-like, *NtNCED3-2* and *NtPIF3*). The present study indicates that JcWRKY is a potential TF facilitating improved photosynthesis with the wax metabolic co-ordination in transgenics during stress.

## Introduction

The light-harvesting process of pigmented, particularly the green organisms, is the support system of life on planet earth. Plants are capable of synthesising organic material for food by utilising carbon dioxide and water in the presence of sunlight via the process of photosynthesis. The understanding of this fundamental process is, therefore, essential to ensure food security and sustained life. Apart from the critical light factor, other factors like salinity, temperature, drought, mineral nutrition, biotic stresses also limit photosynthesis. The different stresses reduce the plant biomass^1^ and thereby, restrict crop productivity. Various kinds of biotic and abiotic stresses impose a limitation on plant’s growth, productivity and survival. Among abiotic stresses, salt stress causes significant agricultural loss, and more than 20% of irrigated land is affected worldwide by salinization^2^. Salinity causes both ionic and osmotic stress^3^. The molecular, biochemical, physiological, and morphological responses, including reactive oxygen species (ROS) homeostasis, changes in epicuticular wax and membrane composition.

Salicylic acid (SA) is an important phytohormone, and exogenous SA application in optimal dose alleviates the damaging effects of stress, it is crucial for photosynthetic performance and facilitates acclimatisation to the changing environment. The co-ordination between ROS producing- and ROS-metabolizing processes maintain the total ROS pool in the cellular environment during stress conditions^4^. ROS are signalling molecules, involved in the plant growth and development and also towards priming acclimatisation responses to stresses^5^. The “self-amplifying feedback loop” concept^6^ is playing a role in maintaining the optimum level of both SA and H_2_O_2_ inside the cell during stress condition by interacting with each other. This concept is based on the interaction between SA and H_2_O_2_, wherein, H_2_O_2_ promotes SA accumulation, and SA enhances H_2_O_2_ production. The SA and ROS interplay, control the transcriptional reprogramming during stress^7^ and also facilitate the light acclimation during photosynthesis^8^. The intricately co-ordinated signalling of SA/ROS during single or combinatorial stress orchestrate the complex regulatory network involving different phytohormones, transcription factors (TFs) and genes and fine-tune different metabolic processes of plants. The SA dependent signalling and epicuticular wax accumulation facilitate plant defence; however, the relation between SA biosynthesis/signalling and cuticular wax accumulation still need to be explored.

TFs regulate various abiotic stress-related genes by interacting with promoter *cis*-elements resulting in abiotic stresses tolerance^9,10^. Some stress-responsive TFs (e.g. myeloblastosis oncogene (MYB), basic leucine zipper (bZIP) and dehydration-responsive element-binding (DREB)), also regulate expression of photosynthesis-related genes. Members of the MYB family (*AtMYB60, AtMYB61, MYB124* and *MYB88*) participate via generating both stomatal and non-stomatal responses^11^. TFs regulate photosynthesis associated genes like chlorophyll A/B-binding protein 2 (CAB2), rubisco synthase 1 A (RbcS 1 A) and rubisco synthase 1B (RbcS 1B)^11^. Heterogeneous lipid bilayer, composed from cutin or suberin and waxes, serves as an interface between plant and the environment. The waxy cuticle reduces excessive water loss and prevent heating by absorption of ultraviolet or blue light but facilitates passage of light to palisade mesophyll cells for photosynthesis. Shine (SHN) and wax inducer1 (WIN1) TFs play a role as transcriptional activators of wax accumulation in Arabidopsis^12,13^. WRKY TFs have been well characterised from a wide variety of plants for both biotic and abiotic stress tolerance^14,15^. Involvement of SA in regulating WRKY expression for biotic stress as both defence-activator and defence-repressor is well studied; however, it is less explored for salinity stress^14^. WRKY family contains 60 amino acid long four-stranded β-sheet WRKY DNA binding domain/s (DBD) and zinc finger motifs, consist of highly conserved WRKYGQK motif at N-terminus. WRKYs are divided into groups based on DBD and zinc finger motifs^16^: group I (2WRKY DBDs), II (single WRKY DBD with different C_2_H_2_ zinc finger), and III (single WRKY DBD with C_2_HC zinc finger). WRKY factors show high binding to a W box (C/T)TGAC(T/C)^16^ of the promoters.

WRKY TFs participate in many aspects of plant innate immunity system, which includes pathogen-associated molecular pattern (PAMP) triggered immunity (PTI), effector-triggered immunity (ETI), basal defence, and systemic acquired resistance (SAR)^17^, and since PTI and photosynthesis responses are integrated^18^, the involvement of JcWRKY in photosynthesis needs to be studied. TaWRKY regulates sucrose phosphate synthase (SPS) gene, facilitating sucrose synthesis in both photosynthetic and non-photosynthetic tissues and also provides salinity tolerance^19^. GsWRKY20 was found to upregulate genes for the wax biosynthesis in transgenic Arabidopsis^20^; also overexpression of OsWRKY89 increased the wax production on the leaf surface of the transgenic rice^21^. The *Jatropha curcas* WRKY (JcWRKY) overexpressing tobacco transgenics have earlier shown improved salinity tolerance via co-ordination of SA signalling and ROS homeostasis^22^ and therefore, in the present study, we aimed to elucidate the role of JcWRKY TF in regulation of photosynthesis parameters, leaf epicuticular wax accumulation, leaf stomata density and gene expression analysis in transgenics during salinity, SA and combinatorial stress (NaCl + SA). To our knowledge, this is the first report of JcWRKY showing the interaction between photosynthesis and wax accumulation in transgenics towards improved salinity tolerance.

## Results

### JcWRKY transgenics show improved photosynthetic response during stress

The response of wild type (WT) and JcWRKY transgenics (L41, L43 and L46) towards NaCl/SA treatments alone and in combination (NaCl + SA) was studied. The gas exchange and the chlorophyll fluorescence related photosynthetic parameters were analysed to decipher the photosynthesis efficiency.

#### Gas exchange related photosynthetic parameters

**Figure 1:**
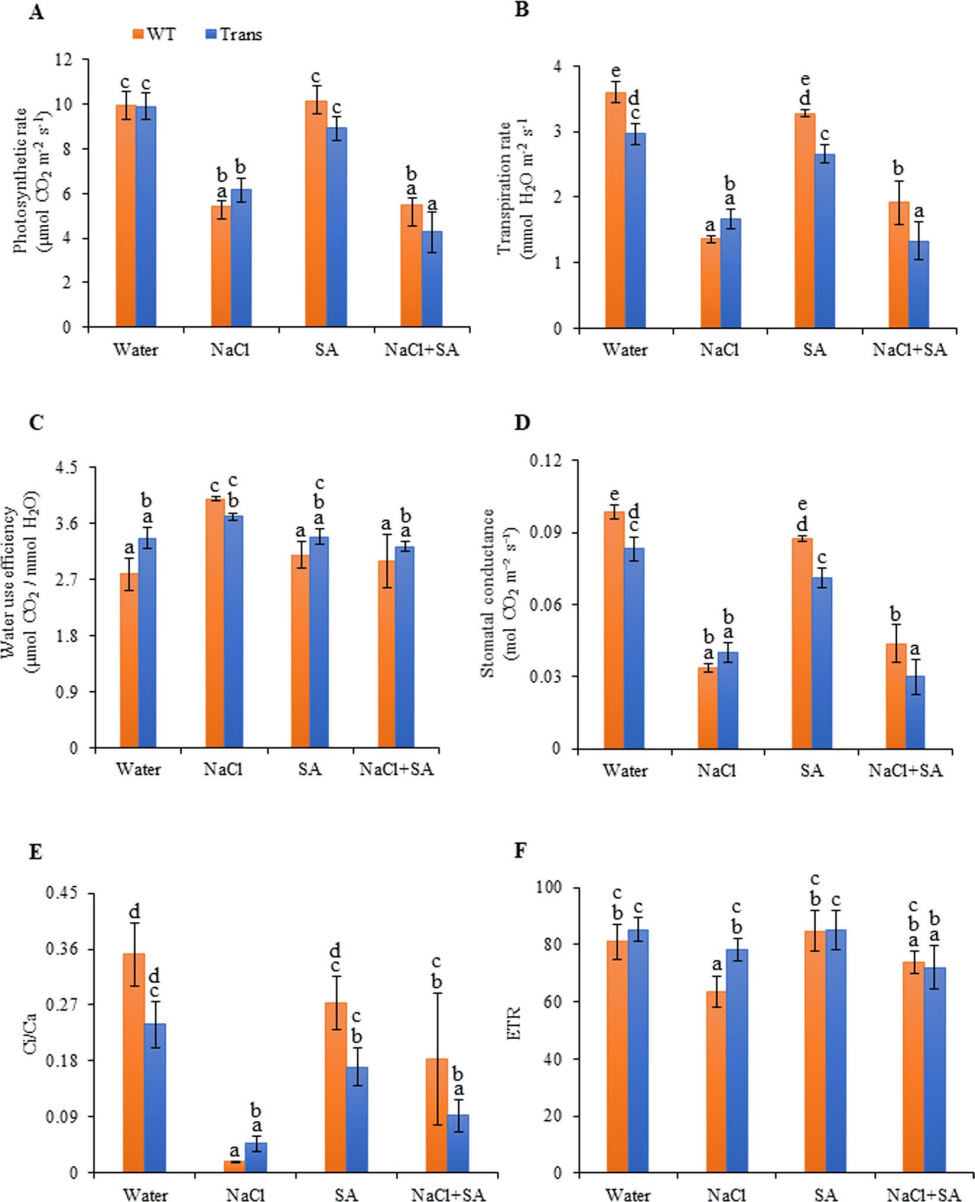
Analysis of gas exchange related photosynthesis parameters (**A**) Photosynthesis rate, (**B**) Transpiration rate, (**C**) Water use efficiency, (**D**) Stomatal conductance, (**E**) Ci/Ca ratio and (**F**) ETR of WT and JcWRKY transgenic lines (average of L41, L43, L46) in response to water, NaCl, SA and combinatorial (NaCl+SA) stress treatments. Values are represented as means ± SD (n = 3) and marked with different alphabets to indicate significant difference at P ≤ 0.05 probability.

The rate of photosynthesis was similar in both WT and transgenics under well-watered conditions (control). The transgenics showed 13% improved photosynthesis rate with NaCl treatment; while with SA and combinatorial NaCl + SA treatment, a decrease of 11.8% and 21.4%, respectively, was observed as compared to WT (Fig. 1A). The transpiration rate of transgenics also showed an increase of 22.3% with NaCl stress and reduced during water (17% lower), SA (18.8% lower) and NaCl + SA (30.5% lower) treatments as compared to WT (Fig. 1B). The water use efficiency (WUE) was higher in transgenics by 20.7%, 9.5% and 7.8% during the water, SA, NaCl + SA, respectively, however, with NaCl stress, it was reduced by 7.09% as compared to WT (Fig. 1C). Stomatal conductance (g) was reduced in transgenics with water (15.6%), SA (18.9%) and NaCl + SA (31.8%) treatments but increased by 19.4% with NaCl stress increased as compared to WT (Fig. 1D). The intercellular CO_2_ concentration/ambient CO_2_ concentration (Ci/Ca) ratio was lower in transgenics with water, SA and NaCl + SA treatments, however, a drastic increase of 170% was observed with NaCl stress as compared to WT (Fig. 1E). The transgenics showed improved electron transport rate (ETR) by 5.3%, 23.36% with water and NaCl, respectively, while in NaCl + SA stress, ETR got reduced by 2.6% as compared to WT (Fig. 1F).

**Figure 2:**
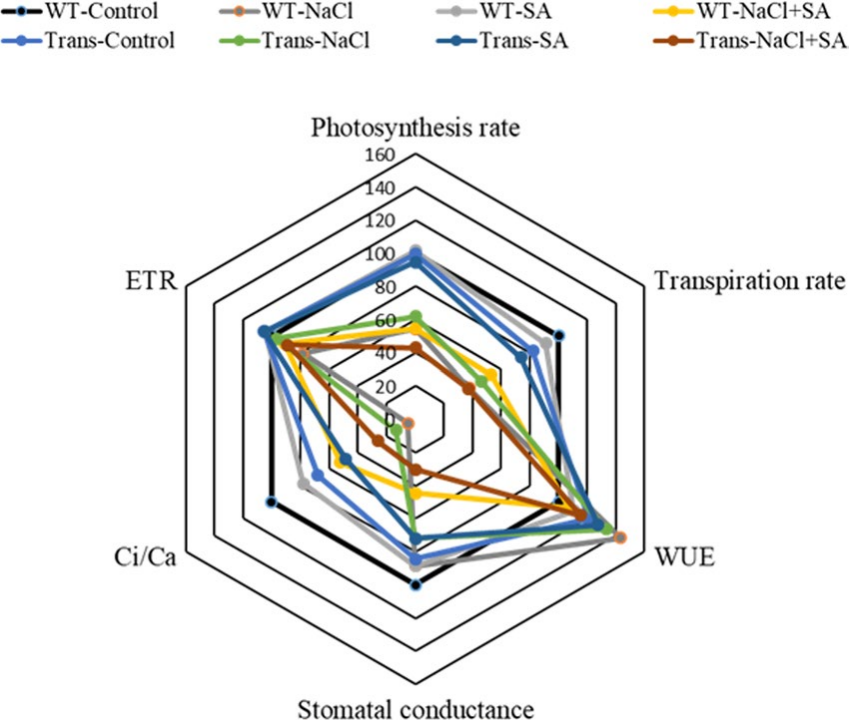
The radar diagram showing comparison of gas exchange related photosynthesis parameters between WT and transgenic plants with water, NaCl, SA and combinatorial (NaCl + SA) stress treatments.

The radar chart was built to visualise the distribution of variation in photosynthetic parameters during control and stress treatments, in transgenics and WT (Fig. 2). WUE was increased during stress conditions in both WT and transgenics as compared to control. However, transpiration rate, stomatal conductance and Ci/Ca ratio were reduced during stress conditions as compared to control (Fig. 2). Ci/Ca ratio was also reduced with salt treatment in transgenics (6-fold) and WT (20-fold) as compared to their respective control conditions.

#### Chlorophyll fluorescence related photosynthetic parameters

Chlorophyll fluorescence was measured simultaneously with an open infrared gas-exchange analyser system equipped with a leaf chamber fluorometer. The parameters like photosynthesis efficiency (Fv/Fm), photochemical quenching (qP), non-photochemical quenching (NPQ), quenching coefficient (1-qP) and quantum yield of PSII electron transport (ΦPSII) were measured in dark-adapted leaves of transgenics and WT with and without stress.

**Figure 3:**
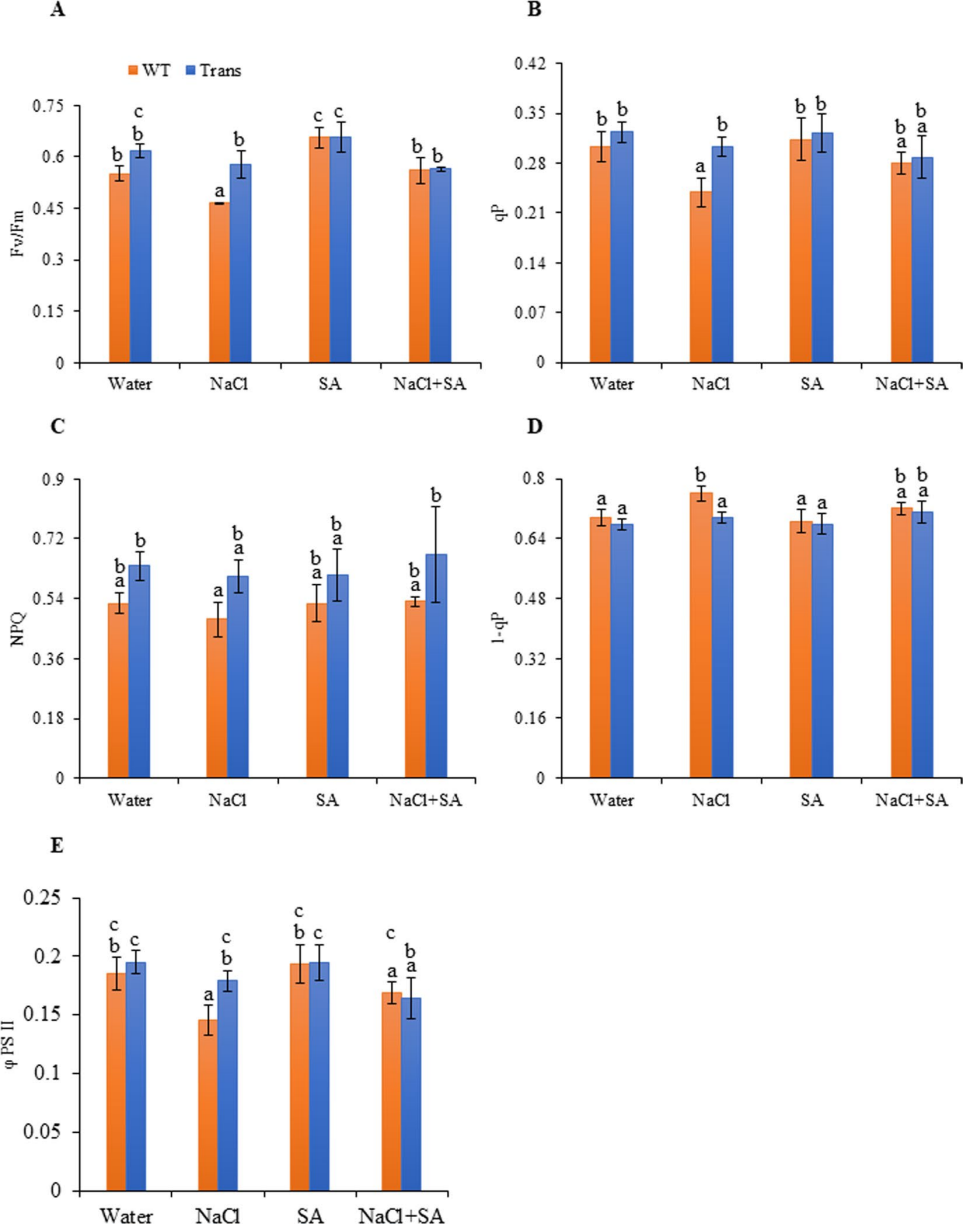
Analysis of chlorophyll fluorescence parameters (**A**) Fv/Fm ratio, (**B**) qP, (**C**) NPQ, (**D**) 1-qP and (**E**) ΦPSII of WT and JcWRKY transgenics in response to water, NaCl, SA and combinatorial (NaCl+SA) stress treatments. Values are represented as means ± SD (n = 3) and marked with different alphabets to indicate significant difference at P ≤ 0.05 probability.

Fv/Fm ratio of transgenics was 11.6% and 24.2% higher during water and salt stress as compared to WT. The Fv/Fm ratio remained unaltered with SA and NaCl + SA stress in both transgenics and WT (Fig. 3A). Higher qP was observed in transgenics with control and all stress treatments as compared to WT. Interestingly, the increase was comparatively higher by 26.8% with salt stress (Fig. 3B). Similarly, transgenics also showed higher NPQ with all stresses, viz. 26.9%, 15.9%, 26.8%, 21.5% higher with NaCl, SA, NaCl + SA and water treatments, respectively, as compared to WT (Fig. 3C). In transgenics, 1-qP was reduced as compared to WT with all treatments, showing a maximum decrease of 8.4% during NaCl stress (Fig. 3D). The transgenics showed higher ΦPSII with water (5.4%) and NaCl (23.2%) and reduced ΦPSII with combinatorial stress NaCl + SA (2.6%) as compared to WT (Fig. 3E).

**Figure 4:**
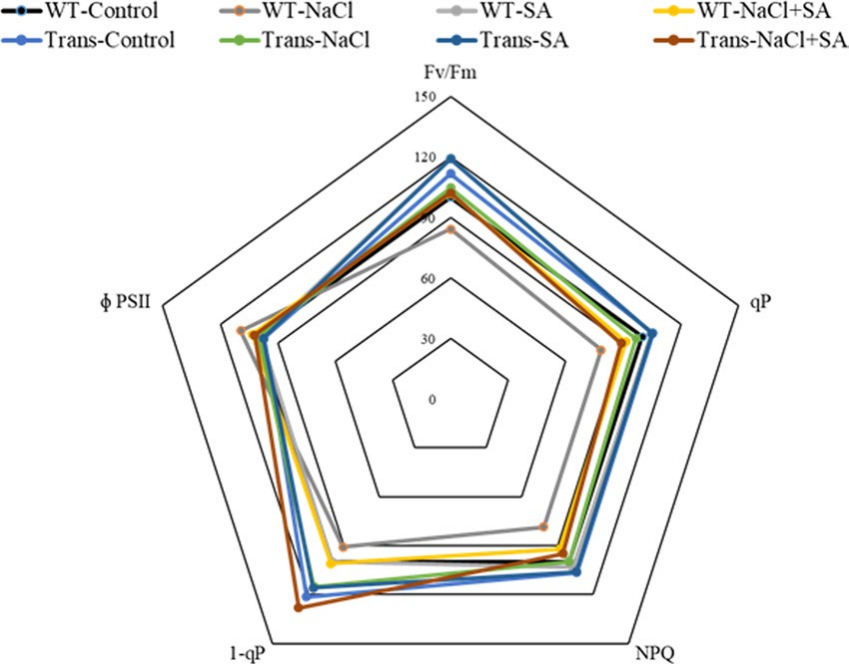
The radar diagram showing comparison of chlorophyll fluorescence related photosynthesis parameters between WT and JcWRKY transgenics with water, NaCl, SA and combinatorial (NaCl + SA) stress treatments.

Radar chart analysis of chlorophyll fluorescence parameters during stress treatments showed that Fv/Fm was significantly decreased in WT with salinity. The ΦPSII showed slight variation in WT and transgenics during all stress treatments. During salinity, WT plants showed ~20% reduction in qP, 1-qP and NPQ, whereas, the transgenics showed better performance. Similarly, 1-qP was better in transgenics (with respect to WT-control) with all treatments, and ~30% increase was detected with NaCl + SA treatment (Fig. 4).

### Differential accumulation of cuticular wax constituents during stress

The quantitative estimation of the epicuticular wax content from the leaves of WT and JcWRKY transgenics in response to different stress treatments was determined using gas chromatography-mass spectrometry (GC-MS) analysis. JcWRKY transgenic showed large variations in the contents of different wax compounds exposed to NaCl and SA stress. Wax compounds were categorised into ten subgroups, i.e. alkane, fatty alcohol, fatty acid, carboxylic acid, alkene, terpene, triterpenoid, aldehyde, terpene alcohol and ketone (Table 1). In total, 278 wax compounds were identified. Among these ten subgroups, alkane was the most abundant identified group as the majority of the compounds, i.e. 143/278 fall within this group, while ketone group was lowest with 1/278 identified compounds (Table 1). Similarly, with respect to the amount of deposited wax on leaves, alkanes were found to be maximum, while ketones showed lowest accumulated wax (Table 2, Supplementary Table S1, terpene alcohol and ketone groups consisting of ≤ 2 compounds were not considered for explanation).

**Table 1:** The number of different wax constituents found in wild type (WT) and JcWRKY transgenic (Trans) in response to water, NaCl, SA and NaCl + SA treatments.

Sr No.	Wax Constituents	WT-Control	Trans-Control	WT-NaCl	Trans-NaCl	WT-SA	Trans-SA	WT-NaCl + SA	Trans-NaCl + SA	Total compounds
1	Alkane	16	18	21	20	13	22	18	15	143
2	Fatty alcohol	5	6	6	5	5	7	6	5	45
3	Fatty acid	6	2	2	2	3	5	8	3	31
4	Carboxylic acid	1	2	2	2	1	3	4	2	17
5	Alkene	3	2	0	0	2	2	4	1	14
6	Terpene	2	0	2	1	1	4	0	2	12
7	Triterpenoid	2	3	1	1	0	0	0	1	8
8	Aldehyde	2	1	1	0	0	1	0	0	5
9	Terpene alcohol	1	0	0	0	0	1	0	0	2
10	Ketone	0	0	0	0	0	1	0	0	1
	Total	38	34	35	31	25	46	40	29	278

**Table 2:** The total amount of different wax constituents (µg/m^2^ leaf area) found in wild type (WT) and JcWRKY transgenic (Trans) in response to water, NaCl, SA and NaCl + SA treatments.

Sr No.	Wax Constituents	WT-Control	Trans-Control	WT-NaCl	Trans-NaCl	WT-SA	Trans-SA	WT-NaCl + SA	Trans-NaCl + SA	Total Amount
1	Alkane	2853.97	2808.65	4057.14	998.088	989.339	2879.37	21641.8	649.569	36877.97
2	Fatty acid	434.921	11.6473	28.5714	7.64818	51.1727	3561.9	1170.21	35.5672	5301.645
3	Fatty alcohol	320.635	282.862	165.714	59.2734	109.168	1676.19	507.092	1506.93	4627.862
4	Carboxylic acid	31.746	26.6223	165.714	30.5927	10.2345	1536.51	2911.35	338.824	5051.59
5	Alkene	263.492	39.9334	0	0	5.11727	126.984	411.348	11.2317	858.1062
6	Terpene	460.317	0	480	3.82409	124.52	790.476	0	56.1587	1915.297
7	Triterpenoid	238.095	81.5308	11.4286	11.4723	0	0	0	5.61587	348.1427
8	Aldehyde	152.381	1.66389	34.2857	0	0	101.587	0	0	289.9179
9	Terpene alcohol	6.34921	0	0	0	0	184.127	0	0	190.4762
10	Ketone	0	0	0	0	0	41.2698	0	0	41.26984
	Total	4761.9	3252.91	4942.86	1110.9	1289.55	9364.02	26641.8	2603.89	53967.88

#### Alkane

**Figure 5:**
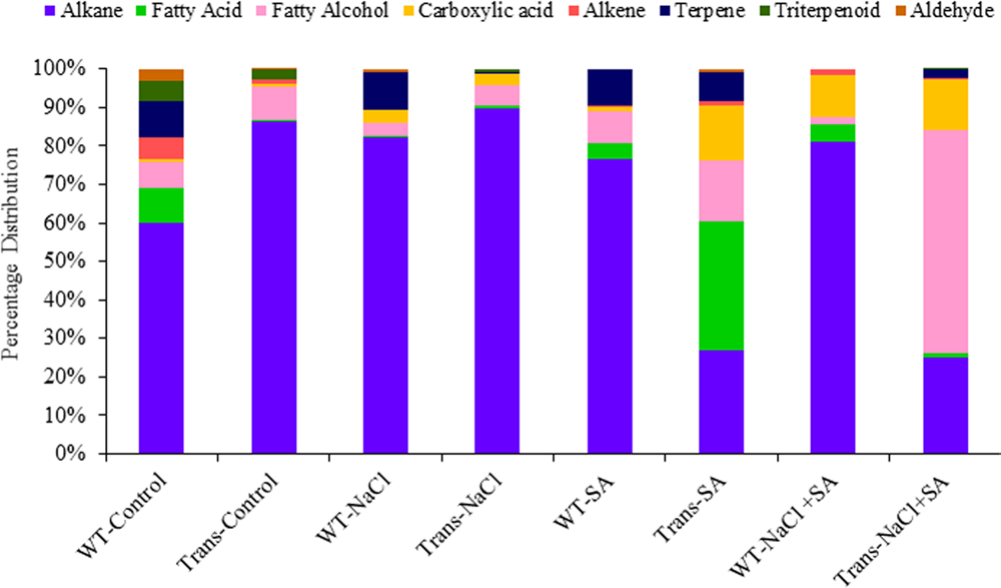
The wax constituent profiling of WT and JcWRKY transgenic (L41, Trans) in response to control, NaCl, SA and combinatorial (NaCl + SA) stress treatments. (For detailed analysis refer Supplementary Table S1).

Among all given treatments, JcWRKY transgenics showed maximum accumulation of alkanes with NaCl stress (90%), and the amount gets decreased with the SA (27%) and NaCl + SA conditions (25%, Fig. 5). The WT plants showed increase accumulation of alkane with NaCl (82%), SA (77%) and NaCl + SA (81%), as compared to control (60%, Fig. 5).

#### Fatty acid

The fatty acid deposition was higher in WT (9%) as compared to transgenic plants (0.4%) during control treatment; however, it increased in transgenics with all stress treatments, showing a maximum increase with SA treatment (33%, Fig. 5).

#### Fatty alcohol

In case of fatty alcohols, both WT and transgenics showed almost comparable quantity with control conditions (WT-7%, transgenics-9%) and NaCl stress (WT-3%, transgenics-5%), however, with SA and NaCl + SA stress, WT plants showed variation, while, transgenic plants showed increased deposition of 16% with SA and 58% with the NaCl + SA (Fig. 5).

#### Carboxylic acid

Carboxylic acid content was maximum in transgenics with SA treatment (14%) and 13% with combinatorial treatment (Fig. 5). In WT plants, it was maximum with combinatorial stress (11%) and 0.8% with SA treatment. The WT and transgenics showed 3.35% and 2.75% of carboxylic acid, respectively, with NaCl treatment (Fig. 5).

#### Alkene

Alkenes were not detected during NaCl stress in both the WT and transgenics. Alkenes were maximum in WT plants during the control condition (6%), but the amount gets drastically reduced with SA (0.4%) and NaCl + SA (2%) stress. While in transgenics, the amount of alkenes was almost similar in control and SA treatments, and further reduced with combinatorial treatment (0.4%, Fig. 5).

#### Terpene

Terpene deposition was almost constant (10%) in WT with control, NaCl and SA. In transgenic plants, no terpene deposition was observed with control treatment; however, 7% and 2% were observed with SA and combinatorial stress, respectively (Fig. 5).

#### Triterpenoid

The WT and transgenics both showed maximum deposition of 5% and 3%, respectively, during control treatment. However, with stress treatments, the triterpenoid deposition was decreased in both WT and transgenic plants (Fig. 5).

#### Aldehyde

Aldehydes were not detected with combinatorial stress in both WT and transgenic plants. Aldehydes showed the highest accumulation in WT plants during control (3%) conditions, and it decreased drastically with NaCl treatment (1%, Fig. 5). While in transgenics, the amount increased (1%) with SA as compared to control (0.1%).

### Scanning electron microscopy of leaf surface during stress conditions

**Figure 6:**
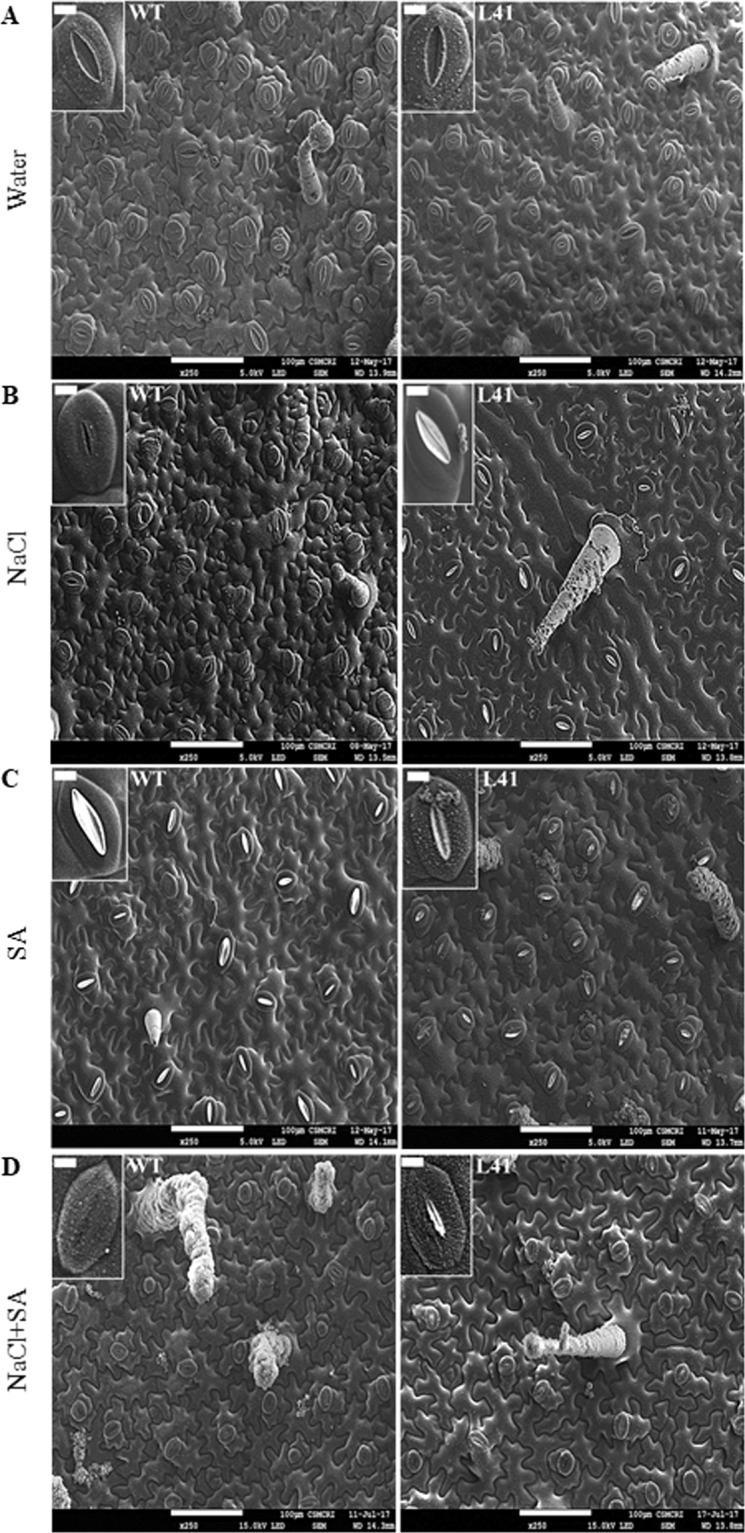
Scanning electron microscopy of the WT and JcWRKY transgenic (L41) abaxial leaf epidermis showing variations in the number of stomata and wax deposition in response to (**A**) water, (**B**) NaCl, (**C**) SA and (**D**) combinatorial (NaCl + SA) stress treatments. The scale bar represents 100 μm. Inset: showing single stomata, scale bar = 10 μm.

**Figure 7:**
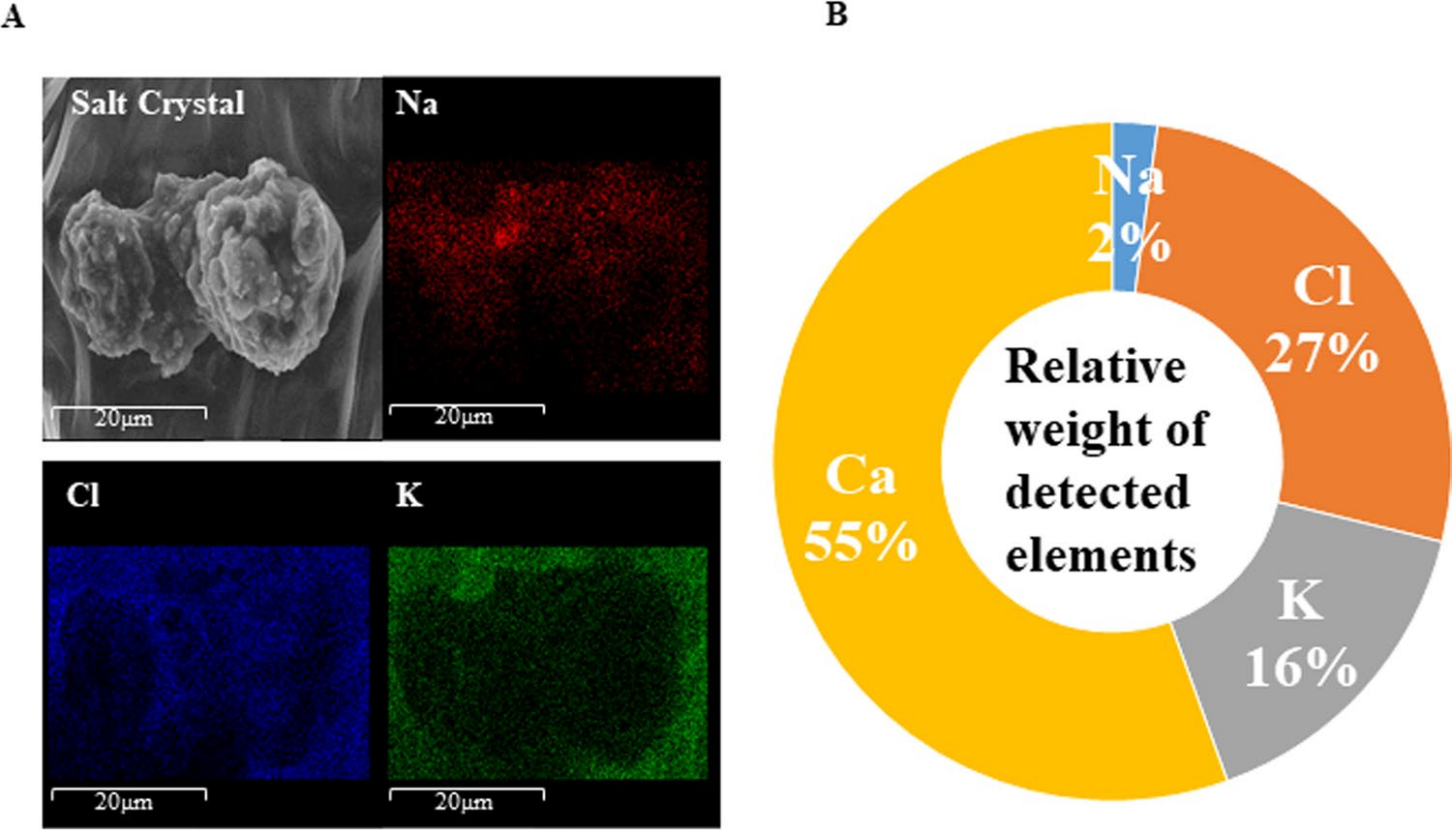
Mapping and EDX analysis of secreted salt crystal on the abaxial leaf surface. (**A**) Na (red colour), Cl (blue colour) and K (green colour) mapping of salt crystal and (**B**) Relative weight (%) of detected elements (Na, K, Ca and Cl). The scale bar represents 20 μm.

Stomata numbers were counted by scanning electron microscopy (SEM) images at 250X magnification of size 475 × 440 μm leaf area. The number of stomata in both WT and transgenics were similar on the abaxial surface, whereas, it was reduced by 3 to 4-fold on the adaxial surface during control conditions (Fig. 6A, Table 3). During stress conditions, the reduced stomatal count was observed in both WT and transgenics. During NaCl stress, transgenics abaxial leaves showed >90% open stomata with some white secretion (Fig. 6B), whereas, WT plants showed higher stomatal count (2-fold, Table 3), with 60% stomatal closure. The SA treatment showed 1.2-fold higher number of stomata on the abaxial surface of transgenics as compared to WT (Fig. 6C), and interestingly, more than 30% stomata were closed in both WT and transgenic plants (Table 3). The WT stomata were slightly elongated and white secretion was observed in both WT and transgenics (Fig. 6C). With SA treatment, adaxial surface showed no difference in the stomatal count (Table 3), and no white secretion was observed in both WT and transgenics (data not shown). With the combinatorial treatment (NaCl + SA), 1.4-fold higher stomatal count was observed in WT as compared to transgenics on both the abaxial and adaxial surface, with >65% of abaxial stomata were closed in both WT and transgenics (Table 3). The white secretion was observed in few stomata (abaxial surface) of transgenics (Fig. 6D). The WT leaves had a higher number of trichomes on both surfaces, whereas, transgenics showed branched trichomes on the abaxial surface. The trichomes on both abaxial and adaxial surfaces were clean (without any deposition) in WT, while transgenics showed the salt crystals deposition. Mapping of salt crystals for Na, K and Cl elements by the field emission-scanning electron microscopy (FE-SEM) revealed that Na and Cl contents were predominantly higher on the leaf surface in the transgenics (Fig. 7A). The energy-dispersive X-ray spectroscopy (EDX) analysis confirmed that the salt crystals were mainly composed of Na, K, Ca and Cl elements (Fig. 7B).

**Table 3:** The stomatal count in wild type (WT) and JcWRKY transgenic on abaxial and adaxial surfaces in response to water, NaCl, SA and NaCl + SA treatments.

Leaf Surface	WT				Transgenic			
		Open	Closed	Total		Open	Closed	Total
Abaxial	Control	32 (±3.6)	1 (±0.7)	33 (±3.5)	Control	32 (±4.0)	1 (±1.5)	33 (±3.6)
	NaCl	13 (±4.2)	20 (±3.3)	33 (±0.9)	NaCl	13(±2.4)	1(±0.6)	14 (±1.5)
	SA	13 (±3.4)	8 (±2.1)	21 (±2.1)	SA	17 (±5.3)	9 (±2.8)	26 (±4.1)
	NaCl + SA	9(±1.2)	19(±1.4)	28 (±2.0)	NaCl + SA	7 (±2.7)	13 (±2.8)	20 (±0.6)
Adaxial	Control	10 (±4.7)	0 (±0.4)	11 (±5.1)	Control	5 (±0.5)	2 (±0.8)	7 (±1.2)
	NaCl	18 (±0)	1 (±0)	19 (±0)	NaCl	10 (±1.4)	1 (±1.2)	11 (±0.5)
	SA	6 (±1.7)	0 (±0)	6 (±1.6)	SA	5 (±1.24)	0 (±0.5)	5 (±1.4)
	NaCl + SA	4 (±1.1)	5 (±0.8)	9 (±0.7)	NaCl + SA	5 (±2.0)	2 (±0.8)	7 (±1.2)

### Differential regulation of downstream genes in transgenics

**Figure 8:**
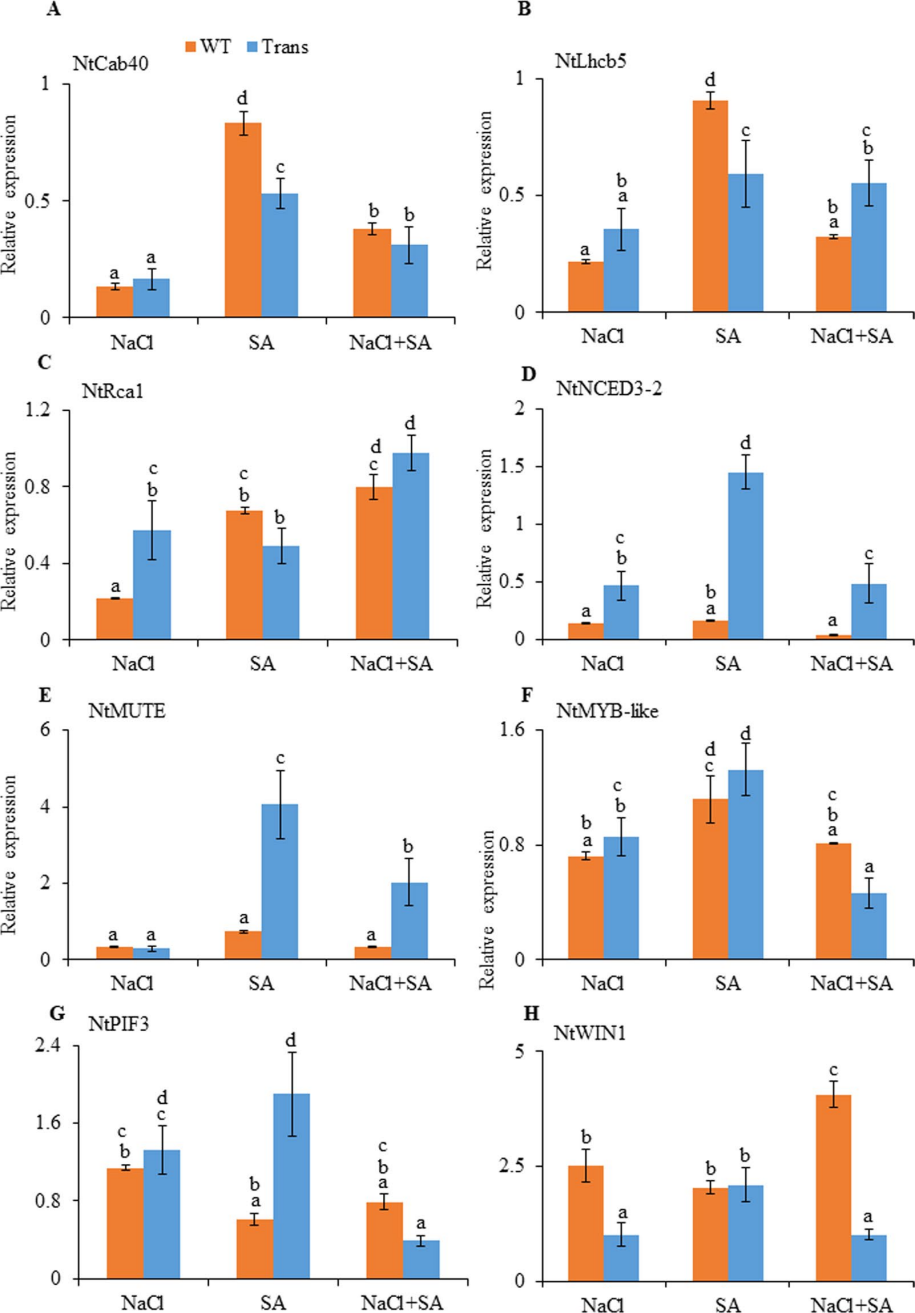
Relative-fold expression of downstream genes (**A**) *NtCab40*, (**B**) *NtLhcb5*, (**C**) *NtRca1*, (**D**) *NtMUTE*, (**E**) *NtMYB-like*, (**F**) *NtNCED3-2*, (**G**) *NtPIF3* and (**H**) *NtWIN1* in WT and JcWRKY transgenics (average of L41, L43, L46) with different stress by Real-time PCR. Values are represented as means ± SE (n = 3) and marked with different alphabets to indicate significant difference at P ≤ 0.05 probability.

The transcript expression of genes related to photosynthesis like *NtCab40* (chlorophyll a/b binding protein 40), *NtLhcb5* (light-harvesting chloroplast pigment-binding protein 5), *NtRca1* (ribulose bisphosphate carboxylase/oxygenase activase 1), genes for wax accumulation *NtWIN1*(wax inducer 1) and stomatal regulation *NtMUTE*, *NtMYB*-like, *NtNCED3*-2 (9-cis-epoxycarotenoid dioxygenase 3-2), *NtPIF3* (phytochrome interacting factor 3) showed differential regulation in WT and transgenics with response to stress treatments. The *NtCab40*, *NtLhcb5* and *NtRca1* transcript were downregulated in transgenics as compared to WT by 56.6%, 52.3% and 38.6%, respectively, with SA treatment (Fig. 8A–C). The transcript of *NtRca1* showed upregulation with NaCl (61.6%) and combinatorial (18.3%) stress (Fig. 8C). The transgenics showed significant downregulation of *NtWIN1* transcript expression in response to salt (148%) and combinatorial stress (300%) (Fig. 8D). JcWRKY transgenics showed increased transcript expression of *NtMUTE* (87.8%), *NtMYB*-like (15.5%), *NtNCED3*-2 (88.7%) and *NtPIF3* (67.5%) with SA treatments (Fig. 8E–H).

## Discussion

Salinity stress alters the hydration as well as the ionic status of the cells, thereby regulating the different physiological and metabolic processes including hormonal changes, reduced enzyme activity and impaired photosynthesis. The WRKY TFs with distinguishable structural features and DNA binding site participate in regulating plant response to growth-development and stresses via auto and cross transcriptional activation and also through post-transcriptional and post-translational regulation. In our earlier work, JcWRKY tobacco transgenics showed salinity and fungal tolerance^22,23^, and in this study, the transgenics showed improved photosynthetic efficiency and wax accumulation during NaCl stress. Although photosynthesis rate was decreased in both WT and transgenics with NaCl treatment, it was comparatively better in JcWRKY transgenics than WT. The decrease in photosynthesis rate due to salt stress was also observed in previous studies by Zhang *et al*.^24^. The increased photosynthesis rate can be attributed to increased Ci/Ca ratio during NaCl stress in transgenics. The Ci/Ca ratio controls the rate of photosynthesis as it portrays the efficiency of CO_2_ consumption^25^. The WUE was improved significantly with both NaCl and NaCl + SA stress as compared to the control conditions, due to decreased transpiration and stomatal conductance during both NaCl and NaCl + SA stress. During stress conditions, the plant closes its stomata, as a protective measure leading to a decrease in water loss, increased stomatal resistance and consequently causing decreased stomatal conductance and transpiration rate leading to improved WUE^26^.

Stomatal conductance was reduced in transgenics during control, SA and NaCl + SA stress. The decreased stomatal conductance might have facilitated the transgenics to reduce transpiration rate and improve WUE. The reduced Ci/Ca ratio in transgenics during control, SA and NaCl + SA stress resulted in decreased photosynthesis rate. Ehleringer *et al*.^25^ reported that a decrease in stomatal conductance leads to reduced Ci/Ca ratio and reduced rate of photosynthesis. Contrastingly, during NaCl stress, stomatal conductance was increased in transgenics which in turn had increased transpiration causing reduced WUE in transgenics than the WT plants, indicating that the behaviour of transgenics during NaCl treatment was different than control, SA and NaCl + SA stress conditions. The ETR, on the other hand, was increased in transgenics than WT to a greater extent with NaCl stress, while it remained unchanged with SA and slightly decreased with NaCl + SA stress, suggesting that SA might be contributing towards normalising the ETR level in both transgenics and WT. The enhanced ETR in salt-tolerant transgenics during salinity stress can be attributed to its efficient/ improved redox homeostasis^22^. Similarly, the salt-resistant wheat cultivar had higher efficiency of electron transport^27^; also the down-regulation of linear electron transport was observed in Arabidopsis; however, an increase in electron flow was found with the halophyte Thellungiella^28^.

Chlorophyll fluorescence measurement is a convenient and non-invasive method; however, it accounts to not more than 1–2% of total light absorbed and gives a critical understanding of the utilisation of excitation energy by PSII and other protein complexes of the thylakoid membranes^29,30^. The chlorophyll fluorescence analysis includes quantification of photochemical and non-photochemical quenching. In general, Fv/Fm, ΦPSII, qP and 1-qP are known to be photochemical-quenching parameters, and NPQ is a non-photochemical-quenching parameter^31^. The fluorescence parameters like Fv/Fm, ΦPSII, NPQ and qP were increased with NaCl stress in transgenics than WT. The Fv/Fm ratio is the maximum quantum efficiency at which light absorbed by PSII is converted to chemical energy^32^. It is a measure of the rate of linear electron transport. Fv/Fm values are generally close to 0.8 during the normal condition in healthy plants and reduce with stress condition, which indicates that a proportion of PSII reaction centre is damaged or inactivated, also known as photoinhibition^33–35^. The Fv/Fm ratio was lower than 0.8 but significantly high in JcWRKY transgenics as compared to WT with NaCl stress, indicating better performance of PSII system in transgenics. The ΦPSII was significantly higher in transgenics than WT with salt stress. The ΦPSII is a parameter for determination of the photosynthetic performance of the plant. During stress conditions, decreased ΦPSII shows accumulation of reduced Qa (a bound quinone) which causes partial damage to primary electron acceptor of PSII, plastoquinone^36^. Reduction of ΦPSII could be explained by the decreased capacity of the carbon metabolism, and/or by low utilisation of ATP and NADPH in a dark phase of photosynthesis^37,38^. The increased value of qP in transgenics with stress condition infers that more light was captured via PSII antennas and more open PSII reaction centres were detected in transgenics, leading to increased efficiency of PSII^39^. In JcWRKY transgenics, NPQ increased during both control and stress conditions, indicating that NPQ was higher in transgenics irrespective of the condition. The dissipation of excitation energy is determined as NPQ of chlorophyll fluorescence during photosynthetic electron transport. During NPQ, the light-harvesting complex (LHC) of PSII undergo conformational changes, thereby generating a modification in pigment interactions causing the development of energy traps. Thus, NPQ protects PSII from photodamage. NPQ is considered as an indicator of excess excitation energy^40–42^. Therefore, the increased values of Fv/Fm, ΦPSII, NPQ and qP during NaCl stress in transgenics, confers that the transgenics were better protected from photosynthetic damage during NaCl stress as compared to WT.

Epicuticular wax participates in providing stress tolerance via regulating leaf temperature, transpiration, WUE etc. In general, alkanes cause 93% increases in total wax amount by increasing the constituents of the long chains (C29, C31 and C33) during dehydration conditions^43^. All the wax groups showed a variable amount of deposition in both WT and JcWRKY transgenics during different stress conditions, suggesting that each type of wax component may be playing a specific role in protecting the plant.

Stomatal density varies in response to external environmental factors such as salinity, drought, temperature, etc. During salinity stress, the number of stomata increased but remain closed in WT plants as compared to JcWRKY transgenics. In strawberry (Fragaria × ananassa), salinity decreases the stomatal number and increases stomatal closure facilitating reduced water loss via transpiration^44^. Also, Karimi *et al*.^45^ showed that a decrease in stomatal cell size is a crucial response during salinity stress in borage. The number of trichomes increased in WT, whereas, the JcWRKY transgenics showed branched larger trichomes. Similarly, increased trichomes were observed in *Atriplex lentiformis*^46^*, Borago officinalis*^47^ and *Schizonepeta tenuifolia*^48^ during salinity stress.

The JcWRKY transgenics showed white deposition at the stomatal aperture and trichomes with all the stress treatments. Fank-de-Carvalho *et al*.^49^ also report white deposition/epicuticular wax particles on stomata and trichomes of *Gomphrena arborescens* leaves. The abaxial leaf surface also showed salt crystal deposition in transgenics. The mapping of salt crystal confirmed the presence of Na and Cl element content. Similarly, salt crystal secretion was also observed by Peng *et al*.^50^ through glandular trichomes in cotton, composed of Na, K, Ca and Cl elements with higher concentrations of Na and Cl elements.

Transcript accumulation of photosynthesis-related genes, *NtCab40*, *NtLhcb5* and *NtRca1*, in JcWRKY transgenics was reduced during SA treatment with respect to WT. In Arabidopsis, the ATHB17 (ARABIDOPSIS THALIANA HOMEOBOX 17) and the HD-ZIP TF reduced the transcription of photosynthesis associated nuclear genes (PhANGs) including light-harvesting complex associated genes and chlorophyll a/b-binding proteins during abiotic stress^51^. The light-harvesting protein complex and chlorophyll-binding proteins capture sunlight/light energy and deliver excitation energy to PSII to facilitate photosynthetic electron transport^52^. The RCA1 is a nuclear-encoded chloroplast protein, involved in carbon fixation during photosynthesis, and promotes plants towards photosynthetic acclimatisation during stress. The JcWRKY transgenics showed higher *NtRca1* transcript regulation with NaCl and combinatorial stress. The rice *RCA* gene shows transcript accumulation during stress conditions^53^. The TF WIN1/SHN1 regulates the biosynthesis of wax, and its overexpression increases the wax content in Arabidopsis^54,55^. The JcWRKY transgenics showed decreased expression of *NtWIN1* with both salt and combinatorial stress, suggesting the regulation and involvement of some other genes for wax accumulation. Stomata play a vital role in the adaptation of plant during abiotic stress. The MUTE gene is involved in the transition from meristemoid to guard mother cell in stomatal biogenesis pathway^56,57^. In transgenics, *NtMUTE* was increased with SA treatment, and the JcWRKY transgenics had a higher number of stomata under SA treatment. The *MYB88* encode MYB proteins that establish stomatal patterning by permitting only a single symmetric division before stomata differentiate^58^. The loss of function of MYB88 resulted in abnormal stomatal spacing and excess stomata development in Arabidopsis^58,59^. In JcWRKY transgenics, higher MYB-like TF expression during NaCl stress could be co-related to the higher percentage of open stomata during NaCl stress, while with combinatorial stress the *NtMYB*-like TF decreased and interestingly, also a decrease in the percentage of open stomata was observed. These results suggest that *NtMYB*-like TF regulates the opening and closing of stomata in JcWRKY transgenics. The significant upregulation of *NtNCED3*-2 and *NtPIF3* in JcWRKY transgenics could be attributed to improved stomatal regulation in transgenics. The *NCED* gene participates in the biosynthetic cascade of abscisic acid (ABA), a hormone involved in regulating the stomata and thereby transpiration. The chromatin immunoprecipitation analysis shows that the Arabidopsis WRKY57 TF binds to *NCED* promoter sequences to regulate its expression^60^. Geilen *et al*.^61^ report that WRKY18 and WRKY40 co-localize with PIFs, including PIF3 (a basic helix-loop-helix TF) to regulate ABA-dependent TF activity.

In conclusion, the present study highlights the role of JcWRKY towards photosynthesis and wax deposition during salinity stress. In the previous study, the tobacco JcWRKY transgenics showed enhanced salinity tolerance via SA mediated ROS homeostasis. Although photosynthesis rate was affected by NaCl stress, the JcWRKY transgenics showed better performance as compared to WT plants. Chlorophyll fluorescence parameters measurement indicated that the photosystem was less damaged in transgenics during stress conditions. The stomatal-count/open-close ratio along with wax accumulation further facilitated transgenic plant’s adaptation to cope up with stress. The GC-MS epicuticular wax analysis identified increased alkane, fatty alcohol, carboxylic acid and fatty acid wax constituents in transgenics on exposure to stress conditions. Thus, our studies on JcWRKY, suggest that JcWRKY intricately co-ordinates signalling of SA/ROS feedback loop during stress and regulates the plants signalling network involving metabolic components co-ordinated by upstream and downstream signalling of different TFs and genes.

## Material and Methods

### Plant stress treatments

The WT and JcWRKY overexpressing T_1_ tobacco transgenics (L41, L43 and L46)^22^ were hardened in a plastic cup containing soil and later transferred to earthen pots in the greenhouse. The one-month-old plants were subjected to 200 mM NaCl, 150 μM SA and combined stress of NaCl and SA (200 mM + 150 μM) every alternate day and the data was recorded after 15 days of the treatment. The leaf tissue of control and stress treated WT and transgenics was collected for real-time gene expression analysis.

### Gas exchange and chlorophyll fluorescence measurement

Photosynthetic gas exchange parameters were measured at photosynthetic photon flux density (PPFD) of 1000 μmol m^−2^ s^−1^ by the open infrared gas analyser (IRGA, Model Li-6400XT, Li-Cor). All other conditions for gas exchange and chlorophyll fluorescence measurement were kept the same as reported in Shukla *et al*.^10^. Photosynthetic gas exchange parameters like net photosynthesis rate, transpiration rate, WUE, stomatal conductance, Ci/Ca ratio, ETR and chlorophyll fluorescence parameters like Fv/Fm, qP, NPQ, 1-qP and ФPSII were recorded after 15 days of treatment.

### Epicuticular wax determination

Leaf samples were collected from stress treated 2-month-old WT and transgenic (L41) for analysis. The leaf samples were immediately immersed in 5 ml of n-hexane and shaken for 1 min at room temperature to extract cuticular waxes. Then n-Hexadecane (C24 alkane, 1 μg/μl) was added as an internal standard. The extracts were filtered and thoroughly dried. Subsequently, derivatisation was done using 100 μl bis-N,N-(trimethylsilyl) trifluoroacetamide (BSTFA, Sigma) for 1 h at 70 °C to transform hydroxyl-containing compounds into their corresponding trimethylsilyl derivatives. Further, the samples were re-dried and re-dissolved in 100 μl of hexane for chemical analysis. The GC-MS analysis was carried out with temperature-programmed on column (DB-5MS) injection and oven temperature set at 60 °C for 2 min, followed by an increase of 15 °C/min to 260 °C and held for 10 min at 260 °C. The temperature was again increased at 5 °C min^−1^ to 320 °C, and held for 15 min. Individual wax components were identified by comparison of their mass spectra with those of authentic standards and as per National Institute of Standards and Technology (NIST) 17 mass spectral library. Quantification was done based on peak areas and the amount of internal standard hexadecane. The total amount of leaf wax components was expressed per unit of leaf surface area.

### Scanning electron microscopy

To examine the leaf wax morphology during control and various stress treatments of WT and transgenic (L41), the samples were attached to the aluminium stubs and coated with gold particles using 90-s bursts from a sputter coater. Samples were investigated using a JSM-7100F field emission SEM (Japan) at an accelerating voltage of 15 kilovolts (kV) and a working distance of 14 mm. The mapping of elements (Na, K and Cl) was carried out in only salicylic acid-treated leaf using EDX for qualitative and relative quantitative analysis. The EDX analysis was carried out for Na, K, Ca and Cl elements from selected leaf surface area and relative amounts of detected elements were expressed as a percentage of the weight of different identified elements.

### Real-time PCR analysis of downstream genes

The quantitative expression of downstream genes involved in photosynthesis, wax accumulation and stomatal regulation was studied by real-time PCR. Total RNA was isolated, and the first-strand cDNA synthesis, qPCR conditions and analysis of control and stress-treated WT and T_1_ transgenic plants were performed as earlier^22^. The primers used in the analysis are mentioned in Supplementary Table S2.

### Statistical analysis

Each experiment was repeated thrice, and the mean values and standard deviations were calculated. Analysis of variance was calculated using Fisher’s least significant difference (LSD) by Infostat software at P ≤ 0.05 to determine the significance of the difference between the means of control and different stress treatments. Mean values of treatments that were significantly different from each other were indicated by different alphabets.

## Supplementary information

Supplementary Information
