# Supplementary material for: The JcWRKY tobacco transgenics showed improved photosynthetic efficiency and wax accumulation during salinity
Source: Sci Rep. 2019 Dec 23;9:19617. doi: 10.1038/s41598-019-56087-6 (PMC6928016; doi:10.1038/s41598-019-56087-6)
Supplement: Supplementary file 1 — Supplementary Information [file 41598_2019_56087_MOESM1_ESM.pdf]

The JcWRKY tobacco transgenics showed improved photosynthetic efficiency and wax accumulation during salinity

Prashant More[1,2], Parinita Agarwal[1,*], Priyanka S. Joshi[1,2], Pradeep K Agarwal[1,2]

[1]Division of Plant Omics,

CSIR-Central Salt and Marine Chemicals Research Institute (CSIR-CSMCRI),

Council of Scientific & Industrial Research (CSIR),

Gijubhai Badheka Marg,

Bhavnagar- 364 002, (Gujarat), INDIA

[2]Academy of Scientific and Innovative Research (AcSIR), Ghaziabad- 201002, India

***Corresponding author**

E-mail address: parinitaa@csmcri.res.in

Telephone +91-278-2564761,

Fax +91-278-2567562

Table S1: List of all the wax compounds identified in GC-MS analysis.

Sr. No.	Compound Group	WT-Control	Trans-Control	WT-NaCl	Trans-NaCl	WT-SA	Trans-SA	WT-NaCl+SA	Trans-NaCl+SA
	**Alkane**								
1	1,1':3',1''-Tercyclopentane, 2'-dodecyl-	-	-	45.71	-	-	-	-	-
2	1,12-Dodecanediol, 2TMS derivative	-	-	-	-	1.71	-	-	
3	10-Methylnonadecane	-	-	-	-	-	-	-	1.87
4	11-Methyltricosane	34.92	9.98	11.43	5.74	52.88	31.75	-	181.58
5	17-Pentatriacontene	-	-	-	-	-	-	340.43	-
6	1-Bromo-11-iodoundecane	-	-	-	5.74	-	-		-
7	1-Chloroeicosane	-	311.15	-		-	-	177.30	-
8	1-Dimethyl(3-chloropropyl)silyloxyoctadecane	-	1.66	-		-	-	-	-
9	1-Hexacosanol	-	4.99	-		-	-	-	-
10	1-Octadecanesulphonyl chloride	-	-	-		3.41	-	-	-
11	2,6,10,14-Tetramethyl-7-(3-methylpent-4-enylidene) pentadecane	158.73	-	-	49.71	-	57.14	60.28	-
12	2,6,10-Trimethyltridecane	-	-	-	3.82	-	-	-	-
13	2-Bromotetradecane	-	-	5.71	-	-	-	-	-
14	2-Butene-1,4-diol, TMS derivative	-	-	-	-	-	60.32	-	-
15	2-Cyclohexylnonadecane	82.54	26.62	51.43	-	39.23	-	-	22.46
16	2-Methylhexacosane	136.51	6.66	28.57	7.65	257.57	133.33	156.03	5.62
17	2-Methylpentacosane	-	-	17.14	-	-	-	-	-
18	4-Methyldocosane	-	-	-	-	-	200.00	-	-
19	5,5-Diethyltridecane	-	-	-	-	-	123.81	-	-

20	Bicyclo[2.2.1]heptane-2,3-dione, 5-(acetyloxy)-1,7,7-trimethyl-, endo-	-	-	-	-	-	50.79	-	-
21	decane, 1-bromo-	-	-	-	51.63	-	-	-	-
22	decane, 1-iodo-	-	1.66	-	-	-	-	-	-
23	decane. 2,3.5.8-tetramethyl-	-	-	-	-	-	-	17.73	-
24	decanedinitrile	-	1.66	-	-	-	-	-	-
25	Dodecane, 1,12-dibromo-	-	-	-	-	-	22.22	-	-
26	Dodecane, 1-cyclopentyl-4-(3-cyclopentylpropyl)-	41.27	-	-	-	-	-	-	-
27	Dodecane, 2,6,10-trimethyl-	-	-	-	-	-	-	-	7.49
28	Dodecane, 2,6,11-trimethyl-	-	3.33	-	-	-	-	-	-
29	Dodecane, 4,6-dimethyl-	-	-	-	1.91	-	-	-	-
30	Dodecane, 4.6-dimethy 1-	-	-	-	-	-	-	31.91	-
31	Dotriacontane	-	-	57.14	-	-	596.83	-	-
32	Eicosane	3.17	6.66	5.71	1.91	-	12.70	24.82	-
33	Eicosane, 2-cyclohexyl-	-	-	-	-	3.41	-	-	-
34	erythro-7,8-Bromochlorodisparlure	139.68	113.14	45.71	3.82	54.58	60.32	393.62	59.90
35	Heneicosane	-	-	-	-	-	-	81.56	-
36	Heptadecane	-	-	5.71	5.74	-	-	-	-
37	Heptadecane, 3-methyl-	111.11	-	-	-	-	-	-	-
38	Heptane, 3,3,5-trimethyl-	-	-	-	-	1.71	-	-	-
39	Hexacontane	453.97	830.28	5.71	15.30	366.74	69.84	453.90	175.96
40	Hexatriacontane	-	-	-	-	-	393.65	-	-
41	Nonacosane	-	-	-	-	-	263.49	-	46.80
42	Nonadecane, 2,6,10,14,18-pentamethyl-	-	-	-	608.03	-	-	-	-
43	Nonadecane, 2,6,10,14-tetramethyl-	-	-	1257.14	-	-	-	-	-
44	n-Tridecan-1-ol	-	-	-	-	-	-	-	18.72
45	Octacosane, 1-iodo-	-	-	-	1.91	-	-	-	-

46	Octadecane	-	-	-	-	-	9.52	24.82	-
47	Octadecane, 3-ethyl-5-(2-ethylbutyl)-	-	-	-	3.82	-	-	-	-
48	Octadecane, 5-methyl-	-	-	17.14	-	-	-	-	-
49	Octane, 2-cyclohexyl-	-	-	-	-	-	-	329.79	-
50	Pentadecane	-	9.98	-	-	-	-	-	58.03
51	Pentatriacontane	-	-	-	-	177.40	126.98	17730.50	-
52	Tetracontane	-	-	-	-	-	234.92	815.60	-
53	Tetracontane-1,40-diol	133.33	113.14	525.71	5.74	-	50.79	92.20	14.98
54	Tetracosane			845.71	-	-	-	819.15	
55	Tetradecane	3.17	1.66	5.71	1.91	1.71	-	-	5.62
56	Tetrapentacontane	536.51	996.67	5.71	5.74	13.65	-	56.74	16.85
57	Tetrapentacontane, 1,54-dibromo-	177.78	356.07	11.43	5.74	15.35	50.79	-	7.49
58	threo-7,8-Bromochlorodisparlure	85.71	-	-	-	-	-	-	-
59	Triacontane, 1-bromo-	673.02	-	1062.86	-	-	-	-	-
60	Triacontane, 1-iodo-	-	-		-	-	253.97	-	-
61	Tridecane, 4,8-dimethyl-	-	-	17.14	-	-	-	-	-
62	Tridecane, 4-cyclohexyl-	-	-	-	-	-	-	35.46	-
63	Tridecane, 6-cyclohexyl-	-	-	-	15.30	-	-	-	-
64	Trispiro[4.2.4.2.4.2.]heneicosane	82.54	13.31	28.57	196.94	-	25.40	-	26.21
65	Undecane, 5-ethyl-5-propyl-	-	-	-	-	-	50.79	-	-
	**Total**	**2853.97**	**2808.65**	**4057.14**	**998.09**	**989.34**	**2879.37**	**21641.84**	**649.57**
	**Percent distributions**	**60.01**	**86.34**	**82.08**	**89.85**	**76.72**	**26.98**	**81.23**	**24.95**
	**Fatty alcohol**								
1	(-)-Isolongifolol methyl ether	-	-	-	-	11.94	828.57	156.03	-
2	11-Methyldodecanol	-	1.66	5.71	1.91	-	12.70	56.74	1.87

3	18-Methyl-nonadecanol, trimethylsilyl ether	-	-	-	-	-	69.84	-	-
4	1-decanol, 2-hexyl-	19.05	13.31	5.71	1.91	8.53	28.57	53.19	3.74
5	1-Dodecanol	-	241.26	-	-	-	-	-	1432.05
6	1-Dodecanol TMS derivative	25.40	-	102.86	15.30	52.88	-	-	-
7	1-Dodecanol. -1-N1S derivative	-	-	-	-	-	317.46	-	-
8	1-Heptatriacotanol	19.05	9.98	-	-	-		-	-
9	1-Hexacosanol	44.44	-	11.43	-	-	-	78.01	67.39
10	1-Octadecanol, TMS derivative	-	-	-	-	-	-	46.10	-
11	1-Pentacosanol	212.70	-	-	-		-	-	-
12	9,19-Cyclolanostan-3-ol, acetate, (3.beta.)-	-	-	-	-	34.12	-	-	-
13	Cholest-5-en-3-ol. (3. alpha.), TMS derivative	-	-	-	-	-	-	117.02	-
14	Docosanol, TMS derivative	-	-	-	-	-	365.08	-	-
15	Docosanol. 1MS derivative	-	1.66	-	-	-	-	-	-
16	Dodecane, 4,6-dimethyl-	-	-	-	-	-	-	-	1.87
17	Isotridecyl alcohol, TMS derivative	-	-	-	-	-	53.97	-	-
18	n-Tridecan-1-ol	-	-	22.86	15.30	-	-	-	-
19	Octacosanol	-	14.98	17.14	24.86	1.71	-	-	-
	**Total**	**320.63**	**282.86**	**165.71**	**59.27**	**109.17**	**1676.19**	**507.09**	**1506.93**
	**Percent distributions**	**6.74**	**8.70**	**3.35**	**5.34**	**8.47**	**15.70**	**1.90**	**57.87**
	**Fatty acid**								
1	.alpha.-Linolenic acid. TMS derivative	-	-	-	-	-	-	166.67	-
2	cis-10-Pentadecenoic acid, isobutyl ester	-	-	-	-	-	22.22	-	-
3	Dibutyl phthalate	-	-	-	5.74	-	-	-	-
4	Doconexent, TBDMS derivative	3.17	-	-	-	11.94	2073.02	205.67	22.46
5	Docosanoic acid, docosyl ester	6.35	-	17.14	-	-	-	-	-

6	Eicosapentaenoic Acid, TBDMS derivat	28.57	-	-	-	32.41	-	-	-
7	Hexacosanoic acid, TMS derivative	6.35	-	-	-	-	-	-	-
8	i-Propyl 11,12-methylene-octadecanoate	-	-	-	-	-	-	35.46	-
9	Lignoceric acid, TMS derivative	-	-	-	-	-	-		7.49
10	Methyl 5,9-tetracosadienoate	-	-	-	-	-	15.87		-
11	Myristic acid, TMS derivative	-	-	-	-	-	-	17.73	-
12	n-Hexadecanoic acid	-	-	-	-	-	-	226.95	-
13	Octadecanoic acid	-	-	-	-	-	-	336.88	-
14	Octadecanoic acid, dodecyl ester	174.60	-	-	-	-	-	-	-
15	Octanoic acid, dodecyl ester	-	-	11.43	-	-	-	-	-
16	Palmitic Acid, TMS derivative	-	-	-	-	6.82	117.46	120.57	-
17	Pentadecafluorooctanoic acid, octadecyl ester	-	1.66	-	1.91	-	-	-	5.62
18	Pimaric acid, TMS derivative	-	-	-	-	-	1333.33	-	-
19	Propanoic acid, 3,3'-thiobis-, didodecyl ester	215.87	-	-	-	-	-	-	-
20	Stearic acid, TMS derivative	-	9.98	-	-	-	-	60.28	-
	**Total**	**434.92**	**11.65**	**28.57**	**7.65**	**51.17**	**3561.90**	**1170.21**	**35.57**
	**Percent distributions**	**9.15**	**0.36**	**0.58**	**0.69**	**3.97**	**33.37**	**4.39**	**1.37**
	**Carboxylic acid**								
1	(S)-Ethyl 3-methyl-5-((4aS,8aS)-2,5,5,8a-tetramethyl-3,4,4a,5,6,7,8,8a-octahydronaphthalen-1-yl)pentanoate	-	-	-	-	-	-	230.50	-
2	[1,1'-Bicyclohexyl]-4-carboxylic acid, 4'-pentyl-, 4-fluorophenyl ester	31.75		80.00	1.91		31.75	159.57	-
3	2-Propenoic acid. pentadecyl ester	-	-	-	-	-	1390.48	2198.58	-
4	Propanoic acid, 3,3'-thiobis-, ditetradecyl ester	-	-	-	-	-	-	-	310.75
5	Propanoic acid, 3-mercapto-, dodecyl ester	-	-	-	-	10.23	-	-	28.08

6	Propanoic acid, decyl ester	-	21.63	85.71	28.68	-	114.29	322.70	-
7	Undecanoic acid, 11-fluoro-, trimethylsilyl ester	-	4.99	-	-	-	-	-	-
	**Total**	**31.75**	**26.62**	**165.71**	**30.59**	**10.23**	**1536.51**	**2911.35**	**338.82**
	**Percent distributions**	**0.67**	**0.82**	**3.35**	**2.75**	**0.79**	**14.40**	**10.93**	**13.01**
	**Alkene**								
1	(-)-Isolongifolol, methyl ether	-	26.62	-	-	-	-	-	-
2	13-Methyl-Z-14-nonacosene	-	-	-	-	-	-	28.37	-
3	17-Pentatriacontene	-	13.31	-	-	-	-	-	-
4	1-Nonadecene	-	-	-	-	-	-	269.50	11.23
5	1-Nonene, 4,6,8-trimethyl-	-	-	-	-	-	-	-	-
6	2,4,6-Tris(cyclohexyl)hept-1-ene	-	-	-	-	1.71	-	-	-
7	3-Hexadecene. (Z)-	187.30	-	-	-		-	-	-
8	3-Octadecene, (E)-	-	-	-	-	3.41	-	-	-
9	9-Hexacosene	31.75	-	-	-	-	28.57	95.74	-
10	cis-1-Chloro-9-octadecene	-	-	-	-	-	-	17.73	-
11	Cyclodecacyclotetradecene, 14,15-didehydro-1,4,5,8,9,10,11,12,13,16,17,18,19,20-tetradecahydro-	44.44	-	-	-	-	98.41	-	-
	**Total**	**263.49**	**39.93**	**0.00**	**0.00**	**5.12**	**126.98**	**411.35**	**11.23**
	**Percent distributions**	**5.54**	**1.23**	**0.00**	**0.00**	**0.40**	**1.19**	**1.54**	**0.43**
	**Terpene**								
1	17.alfa.,21.beta.-28,30-Bisnorhopane	193.65	-	451.43	-	-	92.06	-	39.31
2	17-Pentatriacontene	266.67	-	28.57	3.82	124.52	57.14	-	16.85

3	Andrographolide	-	-	-	-	-	577.78	-	-
4	Dotriacontyl pentafluoropropionate	-	-	-	-	-	63.49	-	-
	**Total**	**460.32**	**0.00**	**480.00**	**3.82**	**124.52**	**790.48**	**0.00**	**56.16**
	**Percent distributions**	**9.68**	**0.00**	**9.71**	**0.34**	**9.66**	**7.41**	**0.00**	**2.16**
	**Triterpenoid**								
1	3-O-Acetyl-6-methoxy-cycloartenol	-	58.24	-	-	-	-	-	-
2	9,19-Cyclolanostan-3-ol, acetate, (3.beta.)-	177.78	-	-	-	-	-	-	-
3	9,19-Cyclolanostan-3-ol, acetate, (3.beta.)-	-	8.32	-	-	-	-	-	-
4	Dotriacontyl pentafluoropropionate	60.32	14.98	11.43	11.47	-	-	-	5.62
	**Total**	**238.10**	**81.53**	**11.43**	**11.47**	**0.00**	**0.00**	**0.00**	**5.62**
	**Percent distributions**	**5.01**	**2.51**	**0.23**	**1.03**	**0.00**	**0.00**	**0.00**	**0.22**
	**Aldehyde**								
1	2-Nonenal, 2-pentyl-	133.33	-	34.29	-	-	-	-	-
2	5-Octadecenal	-	1.66	-	-	-	-	-	-
3	7-Hexadecenal, (Z)-	19.05	-	-	-	-	101.59	-	-
	**Total**	**152.38**	**1.66**	**34.29**	**0.00**	**0.00**	**101.59**	**0.00**	**0.00**
	**Percent distributions**	**3.20**	**0.05**	**0.69**	**0.00**	**0.00**	**0.95**	**0.00**	**0.00**
	**Ketone**								
1	2,6-Dimethyl-9-(1-methylethyl)-1-cyclodecanone	-	-	-	-	-	41.27	-	-

	**Terpene alcohol**								
1	3,7-Dimethyl-2,6-nonadien-1-ol	6.35	-	-	-	-	-	-	-
2	Thunbergol	-	-	-	-	-	184.13	-	-
	**Total**	**6.35**	**0.00**	**0.00**	**0.00**	**0.00**	**184.13**	**0.00**	**0.00**
	**Total Wax**	**4755.56**	**3252.91**	**4942.86**	**1110.90**	**1289.55**	**10673.02**	**26641.84**	**2603.89**

Table S2: List of genes selected for transcript regulation in JcWRKY tobacco transgenics

Sr No	Function of gene	Tobacco gene	Arabidopsis homologue	Primer sequence (5'→ 3')
1.	Photosynthesis	NtCab40/ X52744	AtCAB3/ NM_102731.3	F: CGAGGCTGTATGGTTCAAGGCTG<br>R: AGCCTCTGGGTCTTCAGCAAGT
2.		NtLhcb5/ DQ676845	AtLHCB5/ NM_117102.4	F: GTTGCTATCGCCCCTGCTGACGA<br>R: TAACAAATCCAGCAGCACCGAGC
3.		NtRca1/ NM_001326055	-----	F: TGTTCCTGCTGAAGACGTTGTCA<br>R: TGCAGCCTCTTTGAGGTATTTG
4.	Wax accumulation	NtWIN1/ XM_016591858.1	SHN1/ AT1G15360	F: GTTGCTGAGATTCGTCATCC<br>R: AAGTGAGAGATGGAGAAGGC
5.	Stomatal regulation	NtMUTE/ XM_016620477.1	MUTE/ AT3G06120	F: TTCAAGGAACTTGGAGCATG<br>R: GCTGCTGATGTTGAGATGAAGAA
6.		NtMYB-like TF/ XM_016616074.1	MYB88/ AT2G02820	F: GAGGAGGATGATATACTAAG<br>R: CTTCACAGCATTATCCGTTC
7.		NtNCED3-2/ JX101473	AtNCED3/ NM_112304.3	F: TCATCATCAGAACAGGTTAATTTGG<br>R: TCATCTTCGTTCTCCGAATTTAC
8.		NtPIF3/ XM_016632001	AtPIF3/ NM_001331837.1	F: TATCTGAGCGGAGGCGAAGGGA<br>R: CCCATCGGAGAAAATTGTGGCA
9.	Internal control	NtActin/ XM_016577261.1	-----	F:GATTTGCTGGTGATGATGCTCC<br>R:GTCTCAAACATGATCTGTGTCATC
